# Comparison of Aflatoxin Contamination and Dietary Exposure From Complementary Foods Among Rural Tanzanian Infants Enrolled in the Mycotoxin Mitigation Trial

**DOI:** 10.1002/fsn3.71315

**Published:** 2026-01-30

**Authors:** Rosemary A. Kayanda, Neema Kassim, Erica Phillips, Paul C. Turner, Rebecca Stoltzfus, Francis M. Ngure

**Affiliations:** ^1^ Department of Food Sciences and Biotechnology School of Life Sciences and Bioengineering, The Nelson Mandela African Institution of Science and Technology (NM‐AIST) Arusha Tanzania; ^2^ Division of Nutritional Sciences Cornell University Ithaca New York USA; ^3^ Nutritional Sciences University of Wisconsin‐Madison Madison Wisconsin USA; ^4^ Department of Global Environmental and Occupational Health School of Public Health, University of Maryland College Park Maryland USA; ^5^ Goshen College Goshen Indiana USA; ^6^ Research Consultant Arusha Tanzania

**Keywords:** complementary feeding, contamination, exposure, low aflatoxin flour

## Abstract

Dietary aflatoxins (AF) exposure in early childhood may contribute to growth restriction. The Mycotoxin Mitigation Trial (MMT) was a cluster‐randomized trial designed to assess the effect of providing low‐AF maize and groundnut flours (intervention) on infant growth compared to those consuming typically available flours (standard of care [SoC]). The SoC serves as a control, representing the normal frequency and concentration ranges of AF in this region. MMT initiated at infant age 6 months and ended at 18 months, with the intervention group receiving low‐AF flours monthly throughout. This sub‐study served as one check point in the MMT to assess if there was a difference in AF frequency and concentration in high‐risk foods between the two arms. At the MMT midpoint (infant age 12 months), infant foods were collected during household visits within 20 pre‐selected clusters (10/arm). Maize/groundnut blend and groundnut flours used in the preparation of foods consumed by infants were analyzed for total AFs by ELISA, with 10% confirmed by HPLC. In total 559 foods were sampled; sampling was on one occasion per household. Chi‐square test was used to compare categories of AF contamination in infant foods, and an unpaired *t*‐test was used to compare both contamination by arm, and to compare estimates of AF ingestion between arms. In the intervention arm, 23% of groundnut flour and 6% of blended flour samples had AF levels greater than 10 μg/kg, the legal limit in Tanzania, compared to 45% and 43%, respectively, in the SoC (control) arm (*p* < 0.05). Further, estimated ingestion of AF was lower for the low‐AF supplied blended flours (*p* = 0.03) and groundnut (*p* = 0.04). Importantly, the extremely high levels of AF ingestion (> 1000 ng/kg bw/day) observed in the SoC arm were absent in the intervention arm. The provision of low‐AF flours in the intervention households reduced the frequency and concentrations of AF contamination compared to the SoC, and thus reduced the estimated dietary exposure to infants, at the midpoint of the trial.

## Graphical abstract

The Mycotoxin Mitigation Trial (MMT) followed infants from age 6 months to age 18 months. In one arm all infants were provided with aflatoxin safe flours (maize/groundnut mix and groundnut) for complementary feeding throughout; while the other arm undertook their usual practice, Standard of Care arm. Comparison of estimated aflatoxin intake at the midpoint revealed that the intervention effectively removed the high intake estimates by more than 10‐fold for maize/groundnut mix and 57‐fold for groundnut.

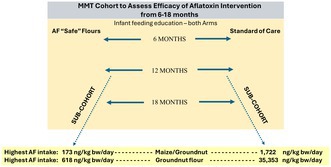

**Abbreviations**
AF: aflatoxin
AFB_1_
: aflatoxin B_1_
ELISA: enzyme linked immunosorbent assay
HPLC: high‐performance liquid chromatography
IQC: internal quality control
KNCHREC: Northern Tanzania Health Research Ethics Committee
LOD: limit of detection
MLs: maximum tolerable limit
MMT: Mycotoxin Mitigation Trial
*n*
: number
SoC: standard of care

## 1 Introduction

Aflatoxins (AFs) constitute a family of toxic and carcinogenic mycotoxins, primarily produced by *Aspergillus flavus* and *A. parasiticus* fungi (Kew 2013; IARC 1993). Of the four naturally occurring AFs, aflatoxin B_1_ (AFB_1_) occurs most frequently and is the most carcinogenic (IARC 1993, 2012). Approximately 500 million people, particularly those residing in developing countries, face a high risk of chronic exposure to dietary AF (Williams et al. 2004). In Sub‐Saharan Africa, the contamination of staple foods with AF is common (Adaku Chilaka and Mally 2020; IARC 2015). The climate, poor soil, and agronomic and post‐harvest practices favor the production and accumulation of AF in tropical regions (Shephard 2003; Wild and Gong 2010). Crops such as maize (corn), groundnuts, cottonseed, and tree nuts are susceptible to AF (Cleveland et al. 2003). AF contamination of common dietary staples contributes to an estimated economic loss, including rejection of produce, reduced food availability and production costs exceeding US$ 750 million in Africa alone (Ostry et al. 2017).

Young children are exposed to AF through complementary foods, typically introduced around 6 months of age when breast milk alone is no longer nutritionally sufficient for growth and development (Dewey 2013). In cross‐sectional and longitudinal studies, AF exposure has been associated with poor linear growth in infants and young children (Gong et al. 2002, 2003, 2004; Lombard 2014; Turner et al. 2007; Watson et al. 2018; Andrews‐Trevino et al. 2021); additionally AF exposure in utero has been associated with poor linear growth from birth to 12 months of age (Turner et al. 2007). In one study of Beninese infants the average height increase from baseline over 8 months was 1.7 cm less on average for those in the highest quartile of AF exposure compared to those in the lowest quartile of exposure, *p* < 0.0001, and remained significant after adjustment for nutritional status (Gong et al. 2004).

In Tanzania, several studies found that young children are frequently exposed to AF through contaminated diets that contain maize and groundnuts (Kamala et al. 2018; Kimanya et al. 2008; Mollay et al. 2022). In two surveys conducted 6 months apart in 25 villages in Kongwa, AF exceeded the legal limit in 18% of maize and 61% of groundnut flours that were used for complementary feeding (Ngure et al. 2023). Additionally, many families in Tanzania initiate complementary feeding earlier than recommended, increasing the risk of exposure at an early age. In Tanzania, 6% of mothers introduced complementary foods at 2 months, and 52% by 6 months (Haruna et al. 2024; Matare et al. 2019).

To provide experimental evidence on the causal‐effect relationship between AF and linear growth we conducted the Mycotoxin Mitigation Trial (MMT), a two‐arm, community‐based cluster randomized trial (Phillips et al. 2020, 2022, 2025). The MMT was designed to isolate the effect of AF exposure by promoting the same infant and young child feeding education to study participants across the two arms, with provision of low AF complementary feeding flours to the intervention arm and promotion of the same flour types and feeding behaviors in the standard of care (SoC) arm, which serves as the control group in that they are using their usual food sources for the flours used for complementary feeding. Our pilot data on this typical dietary behavior informed the main MMT study (Mollay et al. 2021, 2022; Ngure et al. 2023).

Within the main trial, we established a cohort to determine the efficacy of the intervention by including a comparison of diet across the two arms (intervention and SoC), and examining AF contamination of infant‐fed foods (Phillips et al. 2020; Kayanda et al. 2025). The primary objectives of this analysis were (i) to assess AF contamination of foods fed to infants; (ii) to compare the infant food contamination between the two arms of the trial; and (iii) to make estimates of AF intake based on the combined AF contamination, food intake and individual bodyweight of the infants. Analysis of these data provides one critical check‐point to determine the internal validity of the trial, by evaluating the efficacy of the study to create a contrast in AF exposure between the two arms of the MMT.

## 2 Materials and Methods

### 2.1 Study Background and Population

#### 2.1.1 Mycotoxin Mitigation Trial

The MMT took place in the Kongwa District of Dodoma Region, selected due to the high frequency of growth faltering, reliance on smallholder farming practices dominated by maize and groundnut, and a pilot survey confirming AF contamination of cereals used for complementary feeding (Ngure et al. 2023). Infant/mother dyads were recruited into the MMT from health facilities in monthly intervals over 11 months from April 2019 to February 2020.

**FIGURE 1:**
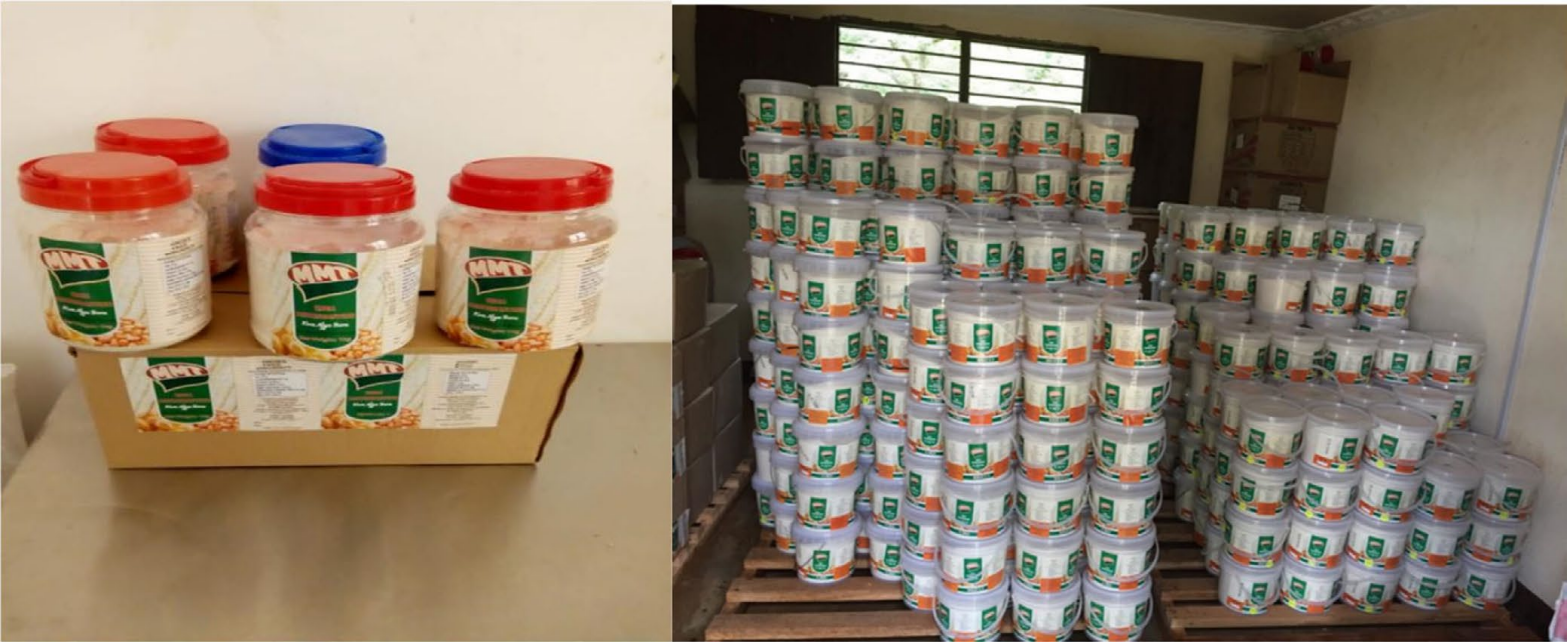
Maize groundnut flours from MMT. Containers of maize/groundnut flour mix 4:1 (locally known as lishe); left 2.5, right 3.5 kg, prepared and supplied by MMT.

The MMT protocol was introduced to the mother when the infant turned 6 months of age and continued until 18 months of age and has been described in depth elsewhere (Phillips et al. 2020, 2025). In brief, participants in both the intervention and the SoC (control) arms were provided with infant feeding education, providing guidance on dietary diversity, feeding frequency and hygiene related to food, and a recommended balance of a 4:1 ratio of maize to groundnut (detailed in Phillips et al. 2025). The intervention arm was supplied with pre‐blended porridge flour made of low‐AF maize and groundnut flour in the ratio of 4:1, and additional low‐AF groundnut flour. These flours were processed through a collaboration with Halisi Products Limited, a local food producer in Arusha, Tanzania (Ngure et al. 2024). The generated low‐AF flours for this study were tested and only transported from Arusha to the research site if the AF concentration was less than 5 μg/kg. This is half the Tanzanian legal limit of 10 μg/kg (IITA 2015), and a conservative decision by the researchers in case of AF proliferation during storage/heterogeneity of the AF in the flours. Flours were produced on a monthly basis for the next month's use, and the quantities distributed each month were calculated to provide all of the caloric needs for infants aged 6–8 months, and progressively fewer total calories and nutrients for older infants as their diets were diversified according to local feeding practices. A 10% buffer ration was added to all packages in case of sharing in the household. The amounts provided were estimated to last approximately 35 days. MMT packages are shown in Figure 1.

#### 2.1.2 MMT Cohort Participant Selection

Within the MMT, we selected a cohort from 20 of the 52 MMT clusters (10 matched pairs per arm) to collect additional, intensive data from. The 20 clusters were selected based on the accessibility of the health centers and households within their catchment areas. As infants recruited into the study at 6 months reached 12 months of age, enrolled mother–child dyads were randomly selected using a random number generator. The 12‐month timepoint was selected because this was the age when most children were consuming complementary food.

Infants were eligible for the study if: (i) they participated in the MMT from 6 months of age (ii) were on average 12 months (range 11–13 months) of age during this sub‐study and, (iii) mothers were willing to provide informed consent for data collection, in addition to the informed consent for the larger MMT trial. Mother‐infant dyads were excluded from this study if they dropped out of the MMT by the time they were visited or did not provide informed consent for additional data collection.

### 2.2 Assessment of Food Intake and Collection of Food Samples

In households that participated in the cohort data collection, we conducted 24‐h dietary recalls with the mother about the child's food intake. From the foods listed in the recall, AF‐susceptible foods were identified and collected by two trained research staff for AF analysis. We sampled maize (250 g), blended maize/groundnut flour (100 g), sorghum and/or finger millet (250 g) and groundnut flour (100 g) (Table 2). In several instances, two distinct samples of the same type of food ingredient were collected from single households. For example, in a household where the index child consumed refined maize flour in “stiff porridge” (locally called *Ugali*) and unrefined, whole maize flour in porridge, each sample was collected and analyzed separately. Compensation ranging from US$ 0.50–0.65 was provided to households for each sample collected, depending on the type of food. Information regarding the source of food (market‐sourced or home‐grown) for the cereals and groundnuts was recorded. Sampling of flours from the intervention arm included those provided by the MMT. If it happened that a child in the intervention arm consumed food acquired by the household, that sample was collected and identified as non‐MMT supplied.

During food sampling, each batch of cereals or flour was mixed by flipping the storage bag or container up and down four times. Four random sub‐samples were drawn from different sections of the stock bag/tin and carefully sealed in a food‐grade, clean zip‐lock bag, providing food collection sizes described above. Air within the zip‐lock bags was expelled during packing, and the bags were securely sealed and stored at −20°C at the MMT lab in Kongwa. Subsequently, all samples were transported on ice to the Nelson Mandela African Institution of Science and Technology (NM‐AIST) laboratory in Arusha and kept at −20°C until analysis.

### 2.3 Aflatoxin Analysis by ELISA and HPLC

Total AF concentrations were quantified using commercially available low‐matrix enzyme‐linked immunosorbent assay (ELISA) kits (Helica Biosystems Inc., USA), following the manufacturer's protocol. AF in maize was analyzed using total AF ELISA (Catalogue number: 941AFL01M‐96), while groundnuts and blended flour were analyzed using a low matrix, total aflatoxin ELISA (Catalogue number: 981AFL01LM‐96). Kit choices were determined by the manufacturer's recommendations for specific food types. The assay standards provided a 1–20 μg/kg range; AF samples exceeding the upper limit were diluted and retested. In each assay, a blank sample and two Quality Control Reference Materials (QCRM) with low AF (3–5 μg/kg) and high AF (13–15 μg/kg) were included. Results for each assay were accepted when both low and high AF‐QCRM samples fell within the specified range. In cases where either the low or high QCRM samples fell out of range, the samples were reanalyzed on a new ELISA plate. Ten percent of the data were confirmed by HPLC using a method described by (Stroka et al. 2000). Sample extraction, clean‐up, and derivatization were conducted before injection into HPLC for aflatoxin detection and quantification. The HPLC detection limit for AFB_1_ and total AF was 0.1 and 0.2 μg/kg, respectively. There was a strong correlation between ELISA and HPLC data, with a correlation coefficient of 0.998 for groundnut flour samples and 0.997 for blended flour samples.

### 2.4 Estimation of Dietary Intake of AFs

The quantities of flour consumed by a child were computed from the amount of porridge consumed, following the estimates of flour content in thin and stiff porridge as described by (Kimanya et al. 2014). Individual exposure of a child to AFs (μg/kg bw/day) was then estimated from the amount of flour consumed (g), the AF contamination level (μg/kg), and the child's body weight (kg).

$$\text{Dietary exposure}\;\left(\mathrm{ng}/\mathrm{kg}\;\mathrm{bw}/\mathrm{day}\right)=1000\times \frac{\text{Flour}\;\left(\mathrm{kg}/\mathrm{day}\right)\times \mathrm{AFs}\;\text{concentration}\;\left(\mathrm{\mu g}/\mathrm{kg}\;\text{in food}\right)}{\text{body weight of the child}\;\left(\mathrm{kg}\right)}$$

### 2.5 Statistical Analysis

For the sample size we estimated that the SD of the AF in any given food type from the control (SoC) would be the same as the mean, and we conservatively predicted a 50% reduction from the intervention. With 95% confidence and 90% power we needed 85 samples per arm. Thus our selection of 142 per arm was sufficiently powered. Data analysis was conducted using STATA 16 (College Station, TZ, USA). A Chi‐square test was used to compare differences in the distribution of AF contamination for each type of food by study arm, using categories of AF < 5 μg/kg a value less than half of the regulation that was preset by MMT as the definition of low AF flour, 5–10 μg/kg as intermediate, and > 10 μg/kg as high, and being above the regulatory limit. An unpaired *t*‐test was also conducted to compare arms; however, since AF was not normally distributed, the data were additionally natural‐log transformed to approximate a normal distribution. All non‐detects were replaced with half the limit of detection. Geometric means and 95% confidence intervals (GM 95% CI) are reported to compare arms for each food group; a *p* ≤ 0.05 was considered statistically significant. Estimated intake by individual food groups and then overall AF intake between arms is additionally reported.

### 2.6 Ethical Clearance

Ethical approval for conducting this research was obtained from Cornell University (Protocol #1809008284) and the Northern Tanzania Health Research Ethics Committee (KNCHREC) with the registration number KNCHREC00041/02/2021. Written informed consent was obtained from mothers before they participated in the study.

## 3 Results

### 3.1 Demographic Information of Participants

**FIGURE 2:**
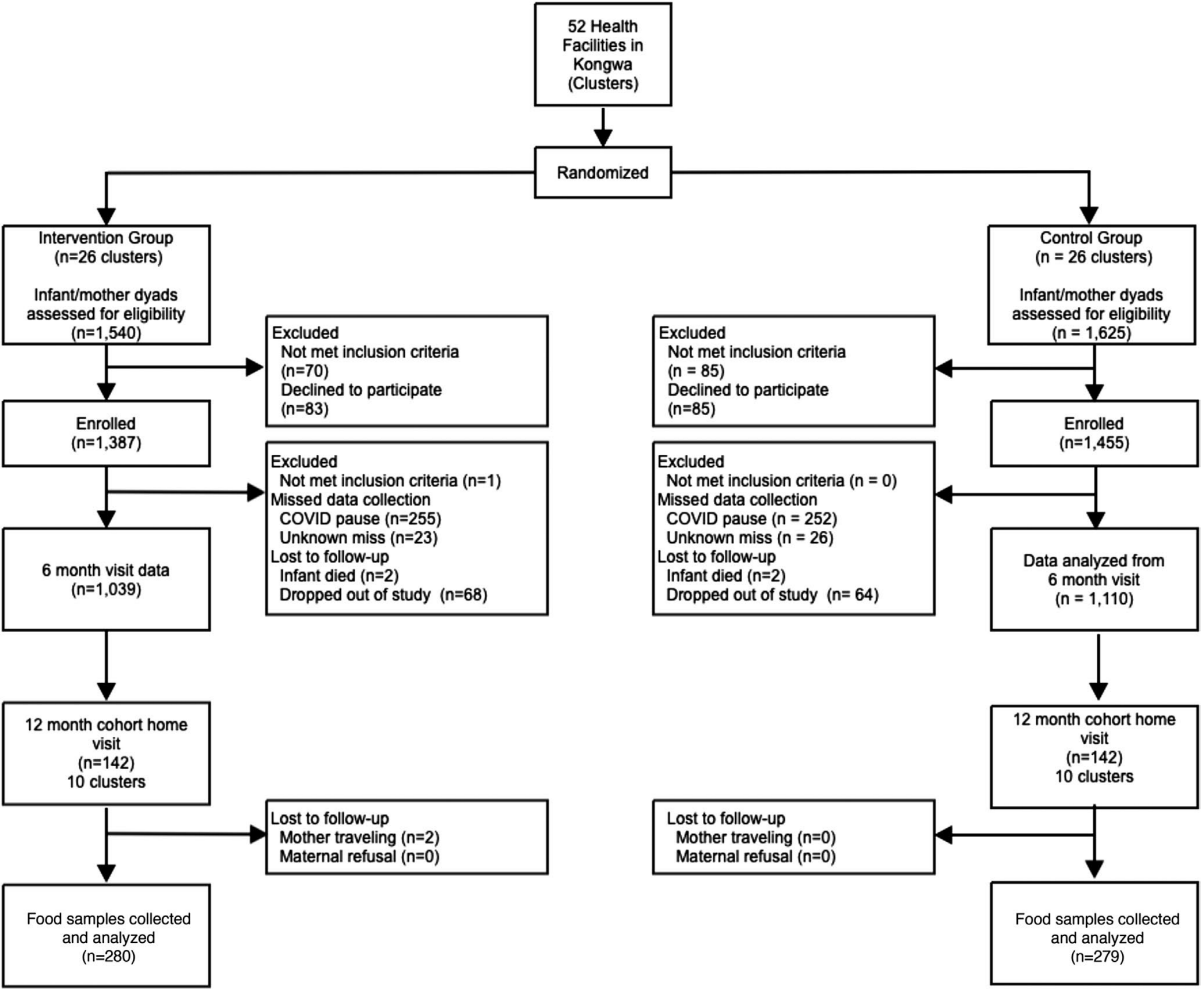
Flow diagram of MMT study and food analysis selection of the sub‐cohort. 284 maternal/child dyads were recruited to assess efficacy of the MMT at the midpoint of the study in terms of creating a contrast in AF exposure between arms. The intervention lost two mothers due to travel, thus overall (*n* = 140 intervention arm and *n* = 142 SoC arm) participants were retained. Each arm provided 280 and 279 food items, respectively for AF analysis.

We recruited 282 maternal/child dyads (*n* = 140 intervention arm and *n* = 142 SoC arm) from within the 20 clusters (Figure 2). This sample size represented approximately 10% of the full trial sample planned for household visits and food sample collection. The demographic characteristics of the mothers were similar between arms. The average age of the recruited infants was ~12 months (Table 1). Almost half (46%) and 44% of the infants in the intervention and SoC arms, respectively, were girls. Most of the mothers were married and above the age of 18 years, with a mean age of 28 years in each arm.

**TABLE 1:** Characteristics of the study of mother‐infant dyads.

Variable	Description	Intervention (*n* = 140)	Standard of care (*n* = 142)
Infant age (months)	Mean (SD, range)	11.7 (0.5, 11–13)	11.7 (0.4, 11–13)
Infant gender, *n* (%)	Males	75 (53.6)	80 (56.3)
	Females	65 (46.4)	62 (43.7)
Breastfeeding, *n* (%)	Currently breast fed	139 (99.3)	138 (97.2)
	No longer breastfed	1 (0.7)	4 (2.8)
Mother's age (years)	Mean (SD, range)	27.8 (7.2, 16–47)	27.9 (7.8, 16–47)
	Married	111 (79.3)	112 (78.9)
Marital status, *n* (%)	Separated/Divorced	13 (9.3)	9 (6.3)
	Single/Never married	16 (11.4)	20 (14.1)
	Widowed	0 (0)	1 (0.7)
People in the house	Mean (SD, range)	5.3 (1.9, 1–11)	5.7 (1.9, 1–11)
	Pit latrine	119 (85.0)	98 (69.0)
Type of toilet, *n* (%)	Ventilated improved pit	20 (14.3)	37 (26.0)
	None, Bush	1 (0.7)	5 (3.6)
	Others	0 (0)	2 (1.4)
Water source, *n* (%)	Protected source	107 (76.4)	108 (76.1)
	Non‐Protected^a^	32 (23.6)	34 (23.9)

^a^ For example, open wells.

### 3.2 Complementary Food Ingredients and AF Levels by Arm

A total of 559 food samples were collected from 282 households (Table 2). Maize, groundnut, and the maize/groundnut blends (or Lishe) were most frequently used and therefore dominated sampling (*n* = 525, 94%) compared to sorghum and millet (*n* = 34, 6%); the former were the focus of this trial. AF was detected in 89/90 SoC blended flours, with 34% at or greater than regulation (range exceeding: 11–244 μg/kg), compared to 100/101 samples with AF detectable in the intervention arm, but only 6% in the intervention arm exceeded regulation (range exceeding: 10–66 μg/kg). The distribution of AF levels in blended flour differed significantly between arms (chi‐squared test *p* < 0.05), Figure S1a. The geometric mean (GM) AF was significantly (*p* = 0.01) higher in the SoC 4.2 μg/kg (95% CI: 3.1, 5.7 μg/kg) compared to the intervention GM 2.8 μg/kg (2.4, 3.2 μg/kg), see Table 3.

**TABLE 2:** Food collection by study arm.

Food type (flour)	Intervention	SoC	Total
Groundnut	72	67	139
Blended^a^	101	80	181
Maize	86	119	205
Sorghum	18	11	29
Finger millet	3	2	5
Total	280	279	559

^a^ Blended maize/groundnut aka lishe.

**TABLE 3:** AF contamination of flours by study arm.

	Blended		Groundnut		Maize^b^	
	Range μg/kg	GM (95% CI) μg/kg	Range μg/kg	GM (95% CI) μg/kg	Range μg/kg	GM (95% CI) μg/kg
SoC	n.d.—244^a^ (*n* = 90)	4.2 (3.1, 5.7)	n.d.—10,206^a^ (*n* = 69)	9.1 (4.8, 17.3)	0.5–785^a^ (*n* = 119)	3.6 (2.6, 4.9)
Intervention	n.d.—66^a^ (*n* = 101)	2.8 (2.4, 3.2)	n.d.—168^a^ (*n* = 72)	3.7 (2.8, 4.9)	n.d.—511^a^ (*n* = 86)	5.8 (4.0, 8.4)
Unpaired *t*‐test		*p* = 0.01		*p* = 0.01		*p* > 0.05

^a^ n.d. non‐detect: blended flour 1 n.d. in each arm; groundnut 5 in SoC, 9 in intervention; maize flour—only one n.d.

^b^ Shaded column—maize only flours were no longer supplied by this mid‐point survey, thus this represents typical household maize flours for both the SoC and intervention, i.e., not a food supplied by MMT.

AF was detected in groundnuts for 64/69 samples in the SoC, with 45% having AF exceeding the AF regulation (range of those exceeding: 11–10,206 μg/kg) compared to 63/72 in the intervention having detectable AF, and 23% exceeding regulation (range exceeding: 11–168 μg/kg). The distribution of AF levels in groundnut flour between arms was significantly different (Chi‐Square test *p* < 0.05), see Figure S1b. AF was significantly (*p* = 0.01) higher in the SoC, GM 9.1 μg/kg (95% CI: 4.8, 17.3 μg/kg) compared to the intervention, GM 3.7 μg/kg (2.8, 4.9 μg/kg), see Table 3. Notably, 13% of groundnut samples in the SoC exceeded 1000 μg/kg, and the range of aflatoxin contamination exceeded 10,000 μg/kg.

The MMT was no longer distributing “maize only” flour for the 12‐month age group; however, home supplies of maize were being used for infant feeding in both arms and thus were sampled. AF was detected in maize for 119/119 samples in the SoC, with 21% in the SoC exceeding the regulatory level (range exceeding: 13–785 μg/kg) compared to maize from the intervention households with 85/86 samples with detectable AF and 34% exceeding the regulation (range exceeding: 11–511 μg/kg). The distribution of AF levels in maize flour between arms for AF was significantly different (Chi‐Square test *p* < 0.05), Figure S1c. However, the GM means were not statistically significantly different; see Table 3.

For sorghum and millet combined (*n* = 34) the GM 0.4 μg/kg (95% CI: 0.3, 0.7 μg/kg) was about 10% of the combined household maize GM (gray column in Table 3). AF contamination in sorghum and millet samples was all below the regulatory limit except for one of the sorghum samples in the intervention arm, which was 16.3 μg/kg. The GM of AF for the intervention arm was 0.4 μg/kg (95% CI: 0.2, 0.8 μg/kg, *n* = 18) was not significantly (*p* = 0.60) different from the SoC arm, GM 0.5 μg/kg (95% CI: 0.2, 1.1 μg/kg, *n* = 11). In millet samples (*n* = 5), AF contamination overall was GM 0.6 μg/kg (95% CI: 0.4, 0.8); no statistical analysis was conducted due to low numbers.

### 3.3 Dietary Exposure to AFs

Dietary intake was first calculated for the three major food items, blended flour, groundnut flour and maize flour, and these data were then used to estimate AF intake from the combined food intake and AF contamination concentration. Dietary behavior is reported as total intake of a food item (mean ± SD in grams (g) and intake per kg bodyweight (means ± SD in g/kg bw)). There was no statistically significant difference in mean food consumption, for each AF food item, between the study arms. For blended flour, the mean intakes of collected samples were 50 ± 20 g (or 5.2 ± 2.3 g/kg bw) for the intervention and 50 ± 27 g (or 5.9 ± 3.2 g/kg bw) in the SoC, *p* = 0.94 and 0.86, respectively. For groundnut it was 34 ± 22 g (or 3.9 ± 2.4) g/kg bw for the intervention and 35 ± 31 g (or 4.1 ± 3.9 g/kg bw) in the SoC, *p* = 0.71 and 0.66 respectively; and for maize flour, it was 37 ± 20 g (or 4.4 ± 2.5) g/kg bw for the intervention and 41 ± 25 g (or 4.8 ± 2.9 g/kg bw) in the SoC, *p* = 0.16 and 0.25 respectively.

Individual intake and household contamination data were used to estimate AF intake for participants in each arm. Estimated AF intake from blended flour and groundnut was significantly higher in the SoC (*p* < 0.025 and *p* < 0.043), respectively, compared to the intervention arm (Table 4). There was no significant difference in estimated AF intake from maize flours (*p* > 0.25).

**TABLE 4:** Dietary exposure to AFs from complementary food ingredients by arms.

Food type	Arm	AF intake range, ng/kg bw/day	% High AF intake, ng/kg bw/day, > 100, > 1000, > 10,000	AF intake, GM (95% CI), ng/kg bw/day	*p*
Blended	Intervention	n.d—173	1 0, 0	14.8 (12.6, 15.8)	0.03
	SoC	n.d.—1722	16, 3, 0	22.6 (2.8, 32.5)	
Groundnut	Intervention	n.d.—618	6, 0, 0	11.9 (8.7, 16.3)	0.04
	SoC	n.d.—35, 353	23, 15, 6	26.3 (12.8, 54.0)	
Maize	Intervention	n.d.—1490	21, 2, 0	15.9 (6.6, 38.0)	0.25
	SoC	1–1729	23, 2, 0	22.1 (11.5, 42.2)	

*Note:* *p*‐value based on unpaired *t*‐test of natural log transformed data. Shaded rows indicate not a food supplied from MMT.

Abbreviation: n.d. not detected.

It was notable that the extremely high consumption of AF, exceeding 1000 ng/kg bw, was eliminated for both the blended flours and the groundnut supplied by the trial for the intervention. Overall intake of AF, where all three food groups were combined to create a composite exposure, did not lead to a statistically significant reduction in estimated AF consumption, driven by the consumption of maize flours in both arms that were not MMT supplied yet contaminated. In the SoC arm, there was a trend to higher levels of AF contamination and consumption in October–December, but was not statistically significant (*p* > 0.05), data not shown. Given the low frequency of sorghum and millet consumption, intake estimates by arm are not reported.

## 4 Discussion

This study measured AF in susceptible foods consumed by a sub‐sample of infants enrolled in MMT. Dietary recalls and food sampling in the households were conducted at the midpoint of the trial when infants were 12 months of age, purposely selected as infants are less reliant on maize/groundnut lishe and are consuming more family foods.

Overall, the provision of low‐AF flours reduced AF levels in food sampled from the intervention households compared to SoC households, providing critical support that the trial succeeded in creating a contrast in AF exposure between arms based on intervention flour use. However, given the frequency of AF contamination in food samples in the intervention arm, it also highlights the difficulty in controlling total AF exposure when household foods are additionally consumed.

The frequency of AF levels above the regulatory limit in Tanzania (10 μg/kg) in blended flour in SoC households was nearly six times higher than in the intervention households. The frequency of levels of AF above the regulatory limit in groundnut flour in SoC households was ~2 times that of intervention households, with 13% of the samples exceeding 1000 μg/kg, and up to 10,205 μg/kg. The levels of aflatoxin in groundnuts and maize in SoC households were, in some cases higher than levels reported in other studies where AF exposure was associated with growth faltering in West Africa (Egal et al. 2005). Generally, households that received low‐AF complementary feeding flours experienced significantly lower AF ingestion compared to households that procured local food ingredients.

Although the provision of low‐AF flours reduced AF exposure, as desired, there were several MMT‐provided food samples from intervention households that had levels above the regulatory limit. This finding was unexpected. Although not investigated here, it is plausible that other food in the households was mixed with our low AF flours or contaminated within the household, as observed elsewhere (Ezekiel et al. 2021; Makori et al. 2019). The approach for food production (Ngure et al. 2024) was extremely rigorous; however it remains difficult to ensure that any batch of food is completely “toxin‐safe” without analyzing the entire food lot being supplied for the MMT study. The removal of estimated exposures with MMT flours in the two highest categories detailed in Table 2 is notable.

Although the intervention arm had lower AF intake and exposure compared to the SoC arm, exposure levels in both arms are of concern. The European Food Safety Authority (EFSA) has set a benchmark‐dose lower confidence limit for a 10% response (BMDL_10_) at 400 ng/kg body‐weight per day for aflatoxin B_1_, and recommends applying a Margin of Exposure (MOE) approach with a target MOE of 10,000 (EFSA 2020). Thus, much of the typical diet in these infants is of concern. The range of AF exposure through blended, groundnuts and maize flours in the non‐intervention arm was higher compared to a previous study in Kongwa (Mollay et al. 2022) and the Rombo District, Tanzania (Kimanya et al. 2014). Dietary exposure to AF in infants was associated with impaired linear growth and stunting in the Chamwino district of the Dodoma region (Makori et al. 2019).

While sorghum and millet were common food ingredients in Kongwa, they were not frequently used for complementary feeding. Mothers reported feeding children these foods only when maize was out of stock. Importantly, these cereals had lower AF levels and were not frequently contaminated. Promotion of sorghum and millet as complementary feed ingredients could therefore reduce the risks of AF contamination and exposure. Furthermore, these crops are highly tolerant to drought, soil salinity, and high temperatures; therefore, they are more relevant in the face of climate change (Sankar et al. 2008).

Key to the internal validity of the MMT was that the randomized intervention could generate a contrast in AF exposure between arms. The present study's strengths include the selection of infants at a time point where complementary foods are starting to dominate, and it captured different months. However, this study was conducted at one age point of the trial which might not be representative of other time points. Food sampling to estimate AF exposure is also not as accurate as using exposure biomarkers (Turner and Snyder 2021); however with a large sample size here, the extremely wide range of contamination reported in the SoC and lack thereof in the intervention provide confidence that the highest levels of exposure are eliminated for intervention flours. In addition, we note that purposive sampling for clusters based on the accessibility of the health facilities limits the generalizability of the findings. However, the study had a wide coverage of 20 clusters out of the 52 in MMT.

## 5 Conclusions

The purpose of this analysis was to verify the efficacy of supplying low‐AF porridge and groundnut flours to reduce AF consumption between the two arms of the MMT study. We found that infant‐consumed food ingredients in Kongwa were frequently contaminated with AF, yet the provision of low‐AF flours reduced average exposures, and perhaps more critically eliminated the highest levels of exposure compared to infants in the SoC. These findings in part support the internal validity of the overall MMT trial, showing that the delivered intervention altered contamination and dietary exposure of children to AF between the intervention and the SoC arms, for those consuming MMT supplied flours only.

## Author Contributions

Rosemary A. Kayanda participated in conceptualization, and methodology, and performed data collection, analysis, interpretation, and writing of initial and consequent drafts of the manuscript. Neema Kassim, Erica Phillips, Paul C. Turner and Francis M. Ngure designed the study and provided a critical review of the manuscript. They also engaged in oversight on lab analysis, data analysis and interpreting the results. All authors have read and agreed to publish this manuscript.

## Funding

This work was supported by the Bill and Melinda Gates Foundation (OPP1155626) through the Mycotoxin Mitigation Trial (MMT) in partnership with the Nelson Mandela African Institution of Science and Technology (NM‐AIST) in Tanzania. PCT was additionally supported by an Institutional Grant from the University of Maryland Grand Challenges Program.

## Conflicts of Interest

The authors declare no conflicts of interest.

## Supporting information


**Figure S1:** (a) Comparison of the percent distribution of AF contamination levels in blended flour samples between arms (intervention: *n* = 101, SoC: *n* = 80). (b) Comparison of the percent distribution of AF in groundnut samples between arms (intervention: *n* = 72 intervention, SoC: *n* = 67). (c) Comparison of the percent distribution of AF in maize samples between arms (intervention: *n* = 86, SoC: *n* = 119 SoC).

## Data Availability

Data supporting the reported results in this paper can be shared upon request with the MMT project. These include the tables of data analyzed and generated during the study. The data that support the findings of this study are available on request from the corresponding author. The data are not publicly available due to privacy or ethical restrictions.
