# Supplementary material for: Comparison of Aflatoxin Contamination and Dietary Exposure From Complementary Foods Among Rural Tanzanian Infants Enrolled in the Mycotoxin Mitigation Trial
Source: Food Sci Nutr. 2026 Jan 30;14(2):e71315. doi: 10.1002/fsn3.71315 (PMC12856770; doi:10.1002/fsn3.71315)
Supplement: Supplementary file 1 — Figure S1: (a) Comparison of the percent distribution of AF contamination levels in blended flour samples between arms (intervention: n = 101, SoC: n = 80). (b) Comparison of the percent distribution of AF in groundnut samples between arms (intervention: n = 72 intervention, SoC: n = 67). (c) Comparison of the percent distribution of AF in maize samples between arms (intervention: n = 86, SoC: n = 119 SoC). [file FSN3-14-e71315-s001.docx]

**Supplemental Figures :** Comparison of Aflatoxin Contamination and Dietary Exposure from Complementary Foods Among Rural Tanzanian Infants Enrolled in the Mycotoxin Mitigation Trial

**Blended Flours – intervention flours from MMT:** The distribution of AF levels in blended flour differed significantly between arms (Chi-square test p < 0.05), Figure S1a. This difference was particularly noticeable in the highest category of contamination, where the SoC had 34% of blended flour samples at >10 μg/kg (range: 11- 244 μg/kg), compared to 6% in the intervention arm (range: 10- 66 μg/kg).

**Figure S1a:** Comparison of the percent distribution of AF contamination levels in blended flour samples between arms (intervention: n = 101, SoC: n = 80).


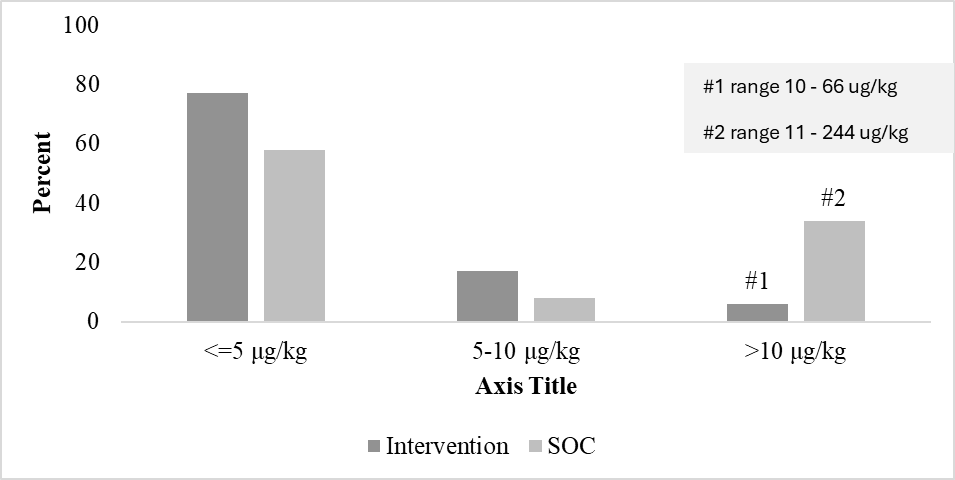


The y-axis represents the distribution of samples within each arm; the x-axis indicates the three categories of contamination described in Materials and Methods. The regulation in Tanzania is 10 ug/kg. The chi-squared test showed a significant difference by arm, p < 0.05. The difference by arm remained significant after adjusting for the cluster effect using multinomial logistic regression.

**Groundnut Flours – intervention flours from MMT:** The distribution of AF levels in groundnut flour between arms was significantly different (Chi-Square test p < 0.05), Figure S1b. Groundnuts from the SoC households were more frequently contaminated with high levels of AF, with 45% having AF at >10 μg/kg (range: 11-10,206 μg/kg) compared to 23% from the intervention arm (range: 11-168 μg/kg).

**Figure S1b:** Comparison of the percent distribution of AF in groundnut samples between arms (intervention: n=72 intervention, SoC: n=67).


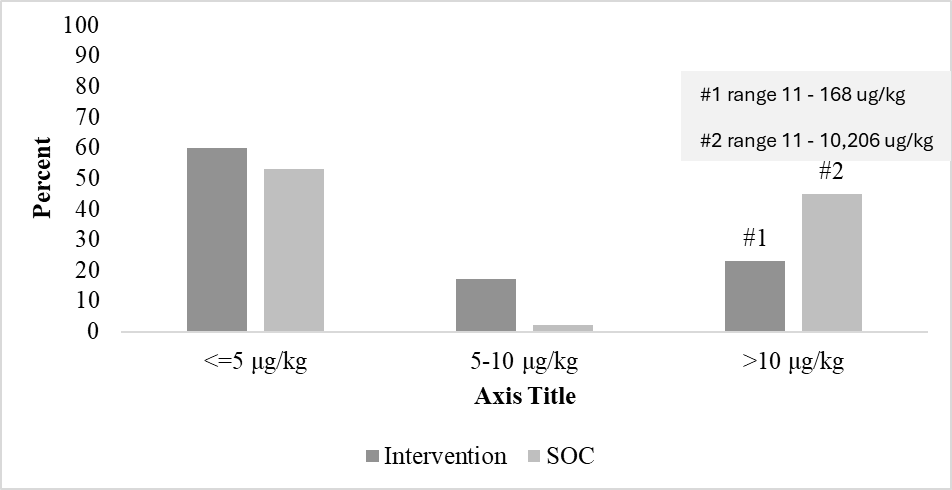


The y-axis represents the distribution of samples within each arm; the x-axis indicates the three categories of contamination described in Materials and Methods. The regulation in Tanzania is 10 ug/kg. The chi-squared test showed a significant difference by arm (p < 0.05). The difference by arm remained statistically significant after adjusting for the cluster effect using multinomial logistic regression.

**Maize Flours – neither arm supplied by MMT**

The distribution of AF levels in maize flour between arms for was significantly different (Chi-Square test p < 0.05, Figure S1c). Maize from the intervention households had frequently higher levels of AF, with 34% having AF at (range: 11 -511 μg/kg), compared to 21% in the SoC arm (range: 13-785 μg/kg). Note: at the time of this survey mothers involved in the current study in the intervention were receiving only maize/groundnut blend and/or groundnut only flours, thus, for both arms, maize flour at this sampling was not provided by the MMT project but was acquired by households and mainly used for Ugali.

**Figure S1c:** Comparison of the percent distribution of AF in maize samples between arms (intervention: n=86, SoC: n=119 SoC).


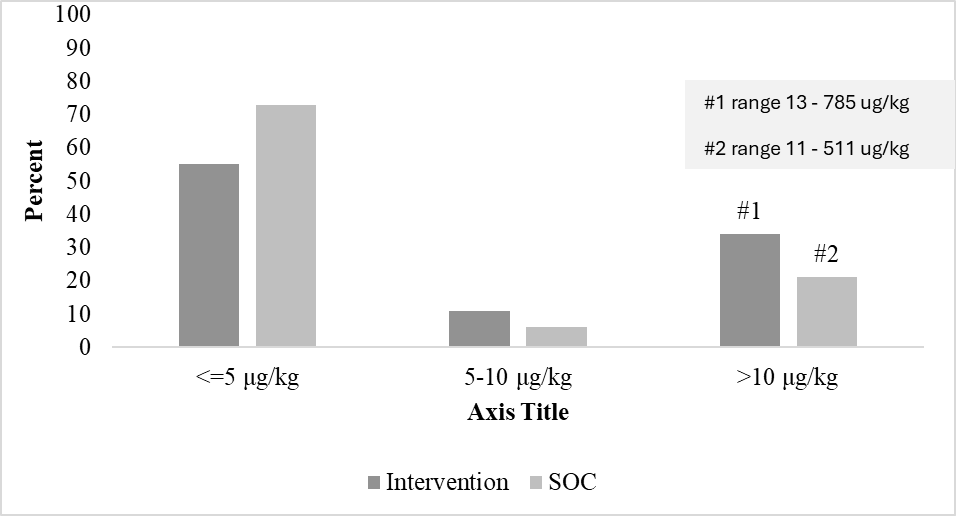


The y-axis represents the distribution of samples within each arm; the x-axis indicates the three categories of contamination described in Materials and Methods. The regulation in Tanzania is 10 ug/kg. The chi-square test showed a significant difference by arm (p < 0.05).
